# COVID-19 knowledge among Ukrainian refugees in Poland: Maria Gańczak

**DOI:** 10.1093/eurpub/ckac131.396

**Published:** 2022-10-25

**Authors:** O Pasek, M Ganczak, A Sikora, E Sobieraj, Ł Duda-Duma, K Pyzio, J Goławski

**Affiliations:** 1 Student Research Group, University of Zielona Gora, Zielona Góra, Poland; 2 Department of Infectious Diseases, University of Zielona Gora, Zielona Góra, Poland

## Abstract

**Background:**

The constantly growing refugee population may constitute public health threat in Poland in the context of COVID-19 pandemic. This study objective was to investigate COVID-19 knowledge among Ukrainian refugees in Poland.

**Methods:**

This cross-sectional study was carried out between March-April 2022 among Ukrainian refugees registering in Zielona Góra, Poland. An anonymous, self-administered questionnaire was used which included 10 questions related to COVID-19 knowledge. Each correct answer was given 1 point.

**Results:**

Response rate was 96.0%, 190 Ukrainians responded (mean age 37.8±15.7 years; 42.1% males); 52.1% were living in the cities >50,000 inhabitants; 61.6% reported high SES; 39% higher education; 44.2% were married. The mean knowledge score was 3.47 (SD ± 2.2), 15.8% collected >50% points. The knowledge level was higher among those with higher SES (p < 0.0001) and higher education (p = 0.003); 31.7% stated that SARS-CoV-2 is an animal-human transmitted disease (more with high SES, p = 0.004), 55.0% considered COVID-19 as highly contagious disease (more living in bigger cities, p = 0.04), 26.3% reported that SARS-CoV-2 infection ensures lifetime immunity (more unmarried, p = 0.02); 24.7% correctly stated that compared with 18- to 30-year-olds COVID-19 mortality rate is about 10 times higher in those who are >65 years (more with higher education and high SES; p < 0.05, p = 0.01 respectively), 44.4% - that COVID-19 treatments are now available (more with high SES and higher education; p = 0.03 both). Regarding prevention, 37.0% reported that FFP3 is the most protective type of mask, 43.3% that vaccines effectively protect against COVID-19 (more with high SES; p = 0.006, p < 0.001 respectively).

**Conclusions:**

COVID-19 knowledge among Ukrainian refugees in Poland was far unsatisfactory, in particular among those with lower education and lower SES. Educational campaigns are urgently needed to effectively raise the knowledge level in this vulnerable group to better control the pandemic.

**Key messages:**

• This study results may be used by public health experts to expand educational campaigns targeting Ukrainian refugees in Poland.

• COVID-19 education oriented to Ukrainian refugees in Poland should specifically address deficits of knowledge identified in this study.

